# Who killed Bruce Lee? The hyponatraemia hypothesis

**DOI:** 10.1093/ckj/sfac071

**Published:** 2022-03-10

**Authors:** Priscila Villalvazo, Raul Fernandez-Prado, Maria Dolores Sánchez Niño, Sol Carriazo, Beatriz Fernández-Fernández, Alberto Ortiz, Maria Vanessa Perez-Gomez

**Affiliations:** Department of Nephrology and Hypertension, IIS-Fundacion Jimenez Diaz UAM, Madrid, Spain; Department of Nephrology and Hypertension, IIS-Fundacion Jimenez Diaz UAM, Madrid, Spain; RICORS2040; Madrid, Spain; Departamento de Medicina, Facultad de Medicina, Universidad Autónoma de Madrid, Madrid, Spain; Department of Nephrology and Hypertension, IIS-Fundacion Jimenez Diaz UAM, Madrid, Spain; RICORS2040; Madrid, Spain; Departamento de Farmacología, Facultad de Medicina, Universidad Autónoma de Madrid, Madrid, Spain; Department of Nephrology and Hypertension, IIS-Fundacion Jimenez Diaz UAM, Madrid, Spain; RICORS2040; Madrid, Spain; Departamento de Medicina, Facultad de Medicina, Universidad Autónoma de Madrid, Madrid, Spain; Department of Nephrology and Hypertension, IIS-Fundacion Jimenez Diaz UAM, Madrid, Spain; RICORS2040; Madrid, Spain; Departamento de Medicina, Facultad de Medicina, Universidad Autónoma de Madrid, Madrid, Spain; Department of Nephrology and Hypertension, IIS-Fundacion Jimenez Diaz UAM, Madrid, Spain; RICORS2040; Madrid, Spain; Departamento de Medicina, Facultad de Medicina, Universidad Autónoma de Madrid, Madrid, Spain; Department of Nephrology and Hypertension, IIS-Fundacion Jimenez Diaz UAM, Madrid, Spain; RICORS2040; Madrid, Spain; Departamento de Medicina, Facultad de Medicina, Universidad Autónoma de Madrid, Madrid, Spain

**Keywords:** Bruce Lee death, cannabis, cerebral oedema, diuretics, heatstroke, hyponatraemia, low osmolar intake, opioids, SIADH

## Abstract

Bruce Lee brought attention to martial arts in the Western world and popularized the quote ‘Be water, my friend’. Lee died at the age of 32 years in Hong Kong on 20 July 1973, under mysterious circumstances. The cause of death is unknown, although numerous hypotheses have been proposed, from assassination by gangsters to the more recent suggestion in 2018 that he died from heatstroke. The necropsy showed cerebral oedema. A prior episode was diagnosed as cerebral oedema 2 months earlier. We now propose, based on an analysis of publicly available information, that the cause of death was cerebral oedema due to hyponatraemia. In other words, we propose that the kidney’s inability to excrete excess water killed Bruce Lee. In this regard, Lee had multiple risk factors for hyponatraemia that may have included high chronic fluid intake, factors that acutely increase thirst (marijuana) and factors that decrease the ability of the kidneys to excrete water by either promoting secretion of antidiuretic hormone (ADH) or interfering with water excretion mechanisms in kidney tubules: prescription drugs (diuretics, non-steroidal anti-inflammatory drugs, opioids, antiepileptic drugs), alcohol, chronic low solute intake, a past history of acute kidney injury and exercise.

‘Be water, my friend’

Bruce Lee, 1940–1973.

## The death of Bruce Lee

Bruce Lee was the most influential martial artist of all time. He inspired millions of people and brought attention to martial arts in the Western world. He was born Lee Jun-fan on 27 November 1940, in San Francisco, California, while his opera singer father was touring the United States. He returned with his parents to Hong Kong as an infant and worked as a child actor. At age 13, he began learning martial arts. At age 18, he returned to the United States where he studied drama and philosophy and opened a martial arts school. At age 26, he first appeared as a martial arts fighter on American television. Lee created his own combat method that he called Jeet Kune Do or the Way of the Intercepting Fist, which combined different training methods such as kung fu, fencing, boxing and philosophy. He popularized philosophical quotes, such as the one starting ‘Be water, my friend…’ [1]. He returned to Hong Kong at the age of 29, where he became a writer, director, lead actor and fight scene choreographer.

Lee died at the age of 32 in Hong Kong on 20 July 1973, under mysterious circumstances. Up to now, the cause of Bruce Lee's death is unknown, although numerous hypotheses have been put forward, from assassination by triad gangsters to the more recent suggestion in 2018 that he died from heatstroke (Table 1) [2, 3].

**Table 1.** Theories about the cause of Bruce Lee's death.

Theories	For	Against
Hypersensitivity to Equagesic	Official cause of death	Not first time of consumption
		No typical salicylate overdose signs/symptoms
		Toxicology report (?)
Assassinated by Mafia (Italian, Chinese, or American)	2 weeks before death, Lee threatened Lo Wei, who was reported to have mobster connections	No evidence of physical injury or poison in autopsy
Family curse	Lee's son, Brandon Lee, died at the age of 28 years: he was shot in a film set when a prop gun fired a live bullet	
	Bruce Lee's older brother died at the age of 3 months under mysterious circumstances	
Cannabis	Necropsy found marijuana in stomach	Oral ingestion is not likely to lead to acute intoxication
Heatstroke	20 July 1973 was reported to be a very warm day	20 July 1973 not warmer than average for summer in Hong Kong Sudden death is unusual No evidence of multiorgan dysfunction in necropsy
	Axillary gland removal 3 months before death^a^	Axillary gland removal not enough to derange thermoregulation. Lee was studied months earlier because of profuse sweating
	Hypothetical prior episode	No evidence of previous episodes
		Profuse sweating in 10 May event
Cocaine use	Lee requested cocaine in writing to Bob Baker	No evidence of cardiovascular comorbidity
		No positive toxicology report announced
Epilepsy	Seizure on 10 May 1973 in the course of prior episode diagnosed as cerebral oedema	No chronic epilepsy No tongue bitemarks On treatment with antiepileptic drugs

^a^According to Polly [3].

## The facts

Some facts about Bruce Lee's death are public [3]. On the day of his death, he and the producer of his films, Raymond Chow, drove to the house of Betty Ting Pei, who was thought to be the mistress of Lee. Lee spent some hours alone with Ting Pei and used marijuana before driving to the house, and while there Lee actively acted out some scenes of an upcoming movie. He experienced headache and dizziness around 7:30 pm, after drinking water. Ting Pei gave him an ‘Equagesic’ pill (a combination of meprobamate and aspirin which he had taken before) and Lee went to the bedroom to rest. Raymond Chow left at that time. At 9:30 pm, Ting Pei found Lee unconscious. She called Chow who went to the house and tried to wake Lee up without success. They called a doctor, who spent another 10 min unsuccessfully doing cardiopulmonary resuscitation. Lee was sent to the nearest hospital, where he was pronounced dead.

At autopsy, there were no signs of external injuries and no tongue bite. Severe cerebral oedema had resulted in brain weight of 1575 g compared with the normal 1400 g [4]. Traces of marijuana were found in the stomach. Bruce Lee's death was officially ruled to be the result of cerebral oedema caused by hypersensitivity to Equagesic.

## Theories about the cause of death

Among the multiple plausible and implausible causes of death, three merit discussion.

**Cerebral oedema caused by hypersensitivity to Equagesic** [5]. Hypersensitivity to the components of Equagesic (aspirin and meprobamate) was identified as the official cause of death [4]. However, Lee had taken this drug before [3] and on the day of his death, he took it AFTER he felt unwell, already having symptoms that may be explained by cerebral oedema (i.e. headache) and cerebral oedema would not be expected to be the only necropsy finding if indeed hypersensitivity to Equagesic was the cause of death.

**Epilepsy**. A seizure was considered a potential cause of the 10 May 1973 episode described below, although as it was a sole episode, a diagnosis of epilepsy was not made. On 29 and 30 May 1973, Lee underwent a full neurologic evaluation: ‘a complete physical, a brain flow study, and an electroencephalogram’, reviewed by neurologist David Reisbord, who did not find abnormalities in his brain functions [3]. Nevertheless, phenytoin was prescribed and Lee was on phenytoin up to the day of his death. Martial artists are at high risk of head trauma or even microtrauma [6]. The most important direct epilepsy-related cause of death is a sudden unexpected death in epilepsy (SUDEP), which is more common in patients with chronic epilepsy [7]. Forensic reports can include tongue bites (20–40%), cerebral oedema and pulmonary oedema [7, 8]. The fact that he had no chronic epilepsy, was on antiepileptic drug therapy and had no tongue bite in necropsy argue against this diagnosis.

**Heatstroke**. This latest hypothesis about the cause of death was proposed by Matthew Polly in his 2018 book *Bruce Lee, A Life* [3].

Heatstroke refers to when body core temperature increases above 40°C, associated with hot dry skin and central nervous system abnormalities [9]. In 2014, the Japanese Association of Acute Medicine (JAAM), published criteria for heat-related illnesses, including heatstroke. A 2016 JAAM Heat Stroke Working Group simplified heatstroke definition to consider patients exposed to high environmental temperature and at least one of the following criteria: (i) Glasgow Coma Scale (GCS) score of ≤14; (ii) creatinine or total bilirubin levels of ≥1.2 mg/dL; (iii) JAAM Disseminated Intravascular Coagulation (DIC) score of ≥4 [10].

Typically, heatstroke results from strenuous exercise (exertional) or from an inability to regulate body temperature (non-exertional), in patients with risk factors such as older age, obesity, heart disease, dementia and others [9]. Initially, heat exposure leading to hyperthermia facilitates the leakage of endotoxin from intestinal mucosa and interleukins from muscles to the systemic circulation, thereby causing a systemic inflammatory response syndrome [10], disruption of the blood–brain barrier and cerebral oedema [11]. Typically, the disorder has three phases resulting in death after 24–96 h, hence sudden death is unusual [12].

Matthew Polly [13] hypothesized that Lee’s removal of axillary sweat glands a month prior to 10 May 1973 could increase the risk of heatstroke. However, sweat is produced through most of the skin surface and sweat output may vary from 0.002 mg/min/gland in the foot's dorsum to 0.016 mg/min/gland in the chest [14]. Thus, it is unlikely that axillary sweat gland removal alone could facilitate heatstroke. Polly further suggested that the 10 May 1973 episode was probably also due to heatstroke [13] and that both days had been uncharacteristically warm for Hong Kong. However, profuse sweating was described during the 10 May episode, despite axillary sweat gland removal [4], the 25°C and 32°C temperatures in both days are common in Hong Kong and there is no evidence that a previous heatstroke episode is a risk factor for a recurrent episode [12]. Overall, the time-course, ability to sweat and lack of extreme weather and of reports by witnesses of excessive heat or skin temperature or dry skin despite a warm environment argue against heatstroke.

## Causes of cerebral oedema

Any hypothesis about the cause of death should account for the necropsy finding of cerebral oedema without further evidence of central nervous system injury and for the acute time-course of the final illness. The four main pathophysiological mechanisms of cerebral oedema are as follows. (i) Vasogenic; commonly seen in conditions with disruption of the blood–brain barrier, e.g. peritumor oedema. (ii) Cellular or cytotoxic; the consequence of brain injury, such as trauma or stroke. (iii) Interstitial oedema resulting from the outflow of cerebrospinal fluid from the intraventricular space to interstitial areas, e.g. hydrocephalus or meningitis. (iv) Osmotic oedema; in which the cells of the brain pull water from the plasma, due to a disequilibrium in osmolarity, e.g. during hyponatraemia [15].

Thus, hyponatraemia stands out as a serious possibility that may account for cerebral oedema and the time-course of the illness, as it is well accepted that acute hyponatraemia itself can be lethal if not treated promptly [16]. Could hyponatraemia-induced cerebral oedema have killed Bruce Lee?

## Water homeostasis

**FIGURE 1:**
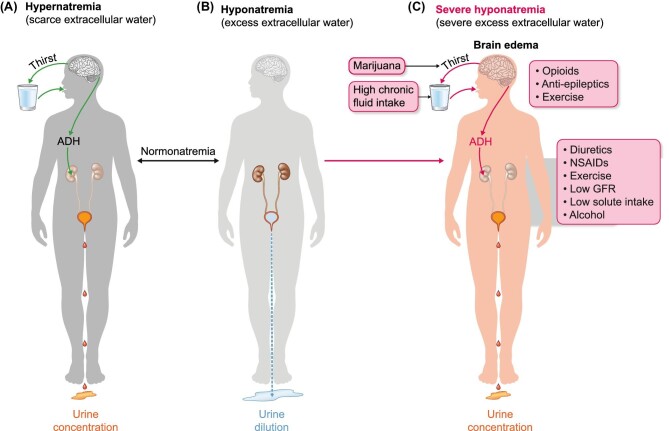
Water balance homeostasis and Bruce Lee's risk factors for hyponatraemia. Hypernatraemia means that there is a deficiency in extracellular water and the body adapts by increasing water intake through thirst and decreasing water loss in urine by secreting antidiuretic hormone (ADH), which results in more a concentrated, lower amount of urine (**A**). If hyponatraemia develops, these mechanisms will be shut down, resulting in an increased volume of more dilute urine (**B**). Severe hyponatraemia may result from failure to timely shut down mechanisms that increase water intake and decrease water excretion once hyponatraemia has developed (**C**). Severe hyponatraemia may trigger cerebral oedema and herniation through the foramen magnum in the occipital bone of the skull, resulting in death within hours. Green arrows indicate adaptive mechanisms. Red arrows indicate mechanisms that are inappropriately activated and not in line with physiological needs to maintain water homeostasis. Lee had several risk factors for severe hyponatraemia that interfered with all levels of control of water balance, from thirst to ADH secretion to ADH action in the kidneys to decrease water excretion in urine.

The balance of water in the body is regulated by plasma osmolarity: increased plasma osmolarity (hypertonicity) is sensed by osmoreceptors in the hypothalamus that regulate both antidiuretic hormone (ADH) release and thirst (Figure 1). Serum sodium concentration (natraemia) reflects water balance. Natraemia is the main contributor to serum osmolarity and an increase in natraemia (and the associated increase in serum osmolarity) will trigger compensatory mechanisms, i.e. thirst, leading to water ingestion and increased secretion of ADH (vasopressin), leading to decreased kidney excretion of water, i.e. a lower volume of more concentrated urine. Conversely, excess body water will decrease natraemia and serum osmolality, leading to the disappearance of thirst and ADH (which has an extremely short half-life) and thus, increased kidney excretion of water in more dilute urine, leading to polyuria and restoring homeostasis. Other forms of water elimination, such as breathing, faeces and sweat, are not regulated. The adaptive capacity of the kidneys is large and in patients with defects in urine concentration (e.g. genetic defects in ADH receptors or aquaporin 2) urine volumes may reach 18–20 L/day. Therefore, it is unusual to develop hyponatraemia (i.e. water intoxication) unless the rate of water intake is clearly superior to this rate of water excretion or there are predisposing factors that limit the kidney’s ability to excrete water. However, this high volume may be surpassed by excessive free water drinking over short periods of time: urinating 20 L/day would translate into being able to handle an excess free water intake of around 0.8 L/h. Acute or hyperacute water loading during ‘dare’ activities involving ingestion of large amounts of water over a short period of time is a cause of cerebral oedema and death within a few hours, even for individuals without risk factors for hyponatraemia. Death due to hyponatraemia leading to cerebral oedema can occur within a few hours of greatly exceeding this rate of water intake, as in recent cases in which 7–8 L of water were ingested within a few hours during ‘dare activities’ [17, 18]. These persons are usually young and healthy. However, even if a reasonable water intake limit is not exceeded, there are factors that limit the kidneys’ ability to excrete a water load, further decreasing the safe amounts of water intake when water intake is not needed. These factors may result in severe hyponatraemia from drinking a much lower water load. Could hyponatraemia have occurred in an apparently healthy young sportsman like Lee?

## Past personal history potentially relevant to the cause of death

The existence of predisposing factors that interfere with water excretion by the kidneys is the most common cause of hyponatraemia and Lee did not appear to have drunk 6–8 L of free water as in some case reports of excessive water ingestion by healthy people. So, did Lee have factors predisposing to hyponatraemia? A brief overview of publicly available information suggests that this is the case.

**Prior cerebral oedema episode.** Lee had suffered a first episode of cerebral oedema 2 months earlier. On 10 May 1973, Lee was in Hong Kong doing a dubbing session. Polly refers to a visit to the bathroom, hash use, headache (‘He felt faint and his head hurt’), disorientation and inability to walk (‘While in the bathroom, Bruce became disoriented and collapsed facedown on the floor’, ‘Sweating star wobbled back to the dubbing room on rubbery legs. The moment he stepped inside the scorching room he fainted again, losing consciousness. Then he vomited his lunch of spaghetti and his body began convulsing’ [3] (Table 2). They took him to Baptist Hospital where cerebral oedema was diagnosed and treated with mannitol. Lee recovered, and on 25 May 1973, he was examined in the United States by Dr. Harold L. Karpman. In his book *Unsettled Matters*, Tom Bleecker states that during the inquest on Lee's death, **‘**Dr Karpman said that blood urea nitrogen (BUN) at Baptist Hospital had been 92 mg/dL, but was normal on May 25 when Karpman examined Lee’ [4]. Thus, Lee appeared to have had acute kidney injury (AKI) and kidney dysfunction is a risk factor for hyponatraemia. A neurologist mentioned that Lee might have an idiopathic seizure disorder and prescribed phenytoin, but epilepsy was not diagnosed as this was an isolated episode. Lee's wife, Linda, stated: ‘Dr Reisbord told me that at no time had Bruce suffered from epilepsy’ [13].

**Table 2.** Lee's symptoms as compared with a prior report of a patient dying from hyperacute hyponatraemia-induced cerebral oedema. Jennifer Strange participated in a radio show contest called ‘Hold your wee for a wii’ and drank 6–7 L of water in under 3 h before dying from severe hyponatraemia [19]. Other examples can be found in a recent review [20].

**Lee**	**Jennifer Strange**
Headache	Headache
‘Feel faint’	‘Feel lightheaded’
Difficulty standing/walking. ‘wobbled back to the dubbing room on rubbery legs’	Difficulty standing/walking; ‘drink more if you guys pick me up’
Vomited	Other contestants vomited
Died within hours	Died within hours

**Excess water intake.** Lee's wife Linda referred to a fluid-based diet (‘carrot and apple juice’). On the evening that Lee died, Polly repeatedly refers to water intake during the day, including just before Lee became noticeably ill. ‘I think we had some water… that probably made him a little tired and thirsty. After a few sips he seemed to be a little dizzy… Immediately after feeling faint, Bruce complained of a headache’ [3]. The excess fluid intake appears to have become a habit for Lee. We suggest that the fact that water intake was repeatedly noticed when it is such a commonplace activity that might have been forgotten given the exceptional circumstances means that it was indeed noticeably higher than the intake of other persons present on the day that Lee died.

**Cannabis.** The use of multiple drugs use by Lee has been reported by Bleecker and multiple sources comment on his frequent uses of hash or marijuana, including on his final day. In July 2021, the English newspaper *The Times*, published a piece of a newly found letter collection written by Bruce Lee: ‘Stoned as hell, but am working on the up-coming character, some coke would help in the formation of what I want to create’ [22]. On the evening that Lee died, Polly repeatedly refers to cannabis use throughout the day. Cannabis causes thirst [23] and may have been driving water intake despite the putative presence of hyponatraemia that we hypothesize. Hyponatraemia has also been linked to cocaine, although reports are uncommon [24]. However, there is no publicly available evidence of cocaine use on the day of his death.

**Alcohol.** Alcohol use has been linked to an increased risk of hyponatraemia. There are reports of increased alcohol intake in the last months of Lee's life: ‘would drink ten to twenty ceramic bottles of sake in an evening.’ ‘Near the end,’ Lowe added, ‘Bruce was often very tired and dizzy’ [4]; his friends say he was drinking alcohol more frequently [3].

**Low dietary solute intake**. The association of excess alcohol intake with hyponatraemia is in part related to a low dietary solute intake. There is evidence that indeed in his last months, Lee’s solute intake was low while water intake was high. An abnormal dietary pattern had been observed by physicians. Bleecker states that ‘Dr Au's advised Bruce to begin eating a balanced and proper diet’ and ‘According to Linda, during her husband's final months, he had stopped eating solid food and was existing miserably on carrot and apple juice’ [4]. This dietary pattern of mostly fluid may have explained the weight loss that appears to have accelerated between May and July 1973. In textbooks, this low solute diet has been labelled as a ‘tea and toast diet’ (Table 3).

**Table 3.** Major causes of hypotonic hyponatraemia [21]. Those that may have applied to Lee, according to publicly available information, are underlined.

Disorders in which ADH levels are not elevated
Polydipsia (e.g. marijuana)
Low dietary solute intake (beer drinker's potomania, tea and toast diet)
Disorders with impaired urine dilution but normal suppression of ADH
Renal impairment
Diuretic-induced hyponatraemia
Disorders with impaired urine dilution due to unsuppressed ADH secretion
Reduced effective arterial blood volume
True volume depletion (hypovolemic hyponatraemia)
Heart failure and cirrhosis (hypervolemic hyponatraemia)
Addison's disease
SIADH (euvolemic hyponatraemia)
CNS disturbances
Malignancies
Drugs: anti-epileptic drugs, opiates, nonsteroidal anti-inflammatory agents
Surgery
Pulmonary disease
Hormonal deficiency: secondary adrenal insufficiency (opiates), hypothyroidism
Hormone administration (vasopressin, desmopressin, oxytocin)
Acquired immunodeficiency syndrome
Disorders with impaired urine dilution due to abnormal V2 receptor (nephrogenic SIADH)
Abnormally low osmostat
Acquired reset osmostat of chronic illness
Genetic reset osmostat
Reset osmostat of pregnancy
Exercise-induced hyponatraemia
Cerebral salt wasting

Bleecker states that Lee complained to Dr. Harold L. Karpman in May 1973 of a poor appetite and that he had lost 20 pounds over a 2-year period. He further adds: ‘In Bruce's particular case, it is important to realize that a loss of twenty pounds of body weight over a two-year period is highly significant. Besides his overuse of diuretics, which significantly reduced his body's percentage of water, Bruce had an extremely low percentage of body fat. The result was that under these conditions a loss of over 14% of his body's muscular mass was noticeable in Bruce and made him appear emaciated…’ And ‘From the day of his near-fatal collapse on May 10 to the day he died nearly ten weeks later, Bruce underwent an inexplicable and startling weight loss’ [4].

Polly indicates, referring to the May event: ‘He had lost 15% of his total body weight in the previous two months, and he had minimal body fat to start’; ‘But to me, he seemed awfully run down. In all the years I knew him, I never saw him in such an emaciated condition before.’ ‘Yeah, I've lost a lot of weight from working day and night,’ Bruce explained… ‘I even forget to eat’ [3].

**Prescription drugs**. Prescription drugs may be the primary cause of severe hyponatraemia [25]. However, the molecular mechanisms of the contribution of some drugs to hyponatraemia have not been completely clarified or may involve several mechanisms. Bleecker writes that during the inquest, ‘he quickly became bored with the complex scientific analysis of the wide assortment of prescription drugs Bruce had been taking’ [4]. This points to the use of other prescription drugs that may have had an impact over predisposition to hyponatraemia. These included diuretics, phenytoin and painkillers (e.g. opioids and non-steroidal anti-inflammatory drugs, NSAIDs). Lee was taking phenytoin and Doloxene (dextropropoxyphene, an analgesic in the opioid category, apparently in combination with aspirin) [3].

**Diuretics.** Diuretic use may have contributed to both AKI and hyponatraemia. Bleecker [4] states that ‘Bruce was a frequent user of diuretics. Although this greatly reduced the excessive water content in his muscles, and thus gave him the desired ‘ripped’ appearance, it was a dangerous practice that frequently placed him in a life-threatening state of dehydration’ [4]. While we have no clear idea of what diuretics were involved, thiazides are known to cause hyponatraemia, while loop diuretics increase free water clearance in persons with normal kidney function. However, even loop diuretics favour hyponatraemia primarily due to hypovolaemia-induced release of antidiuretic hormone (ADH), especially if water intake is high and distal delivery is very low, as may occur during diuretic-induced kidney dysfunction [26].

**Opioids**. We found no report on dextropropoxyphene and hyponatraemia, but other opioids, such as tramadol may cause hyponatraemia [27]. Opioids may promote ADH secretion but may also favour hyponatraemia when ADH is absent [28]. Additionally, they may suppress adrenocorticotropic hormone, favouring secondary adrenal insufficiency, another cause of hyponatraemia [29]. Opioids are mostly associated with hyponatraemia in combination with other drugs predisposing to hyponatraemia [30, 31].

**NSAIDs**. NSAIDs decrease the inhibitory effect of prostaglandins on the activity of ADH, favouring hyponatraemia under conditions of non-suppressible ADH such as the syndrome of inappropriate antidiuretic hormone secretion (SIADH) or in the presence of volume depletion (as may be expected in diuretic users), and increase the risk of hyponatraemia in patients on diuretics [18]. However, aspirin is among the NSAIDs with a lower impact on natraemia.

**Antiepileptic drugs.** Diverse antiepileptic drugs can predispose to hyponatraemia. For newly initiated antiepileptic drugs, adjusted odds ratios (95% confidence interval) for hospitalization due to hyponatraemia, compared with controls, have been reported to be 9.63 (6.18–15.33) for carbamazepine and 4.83 (1.14–25.76) for phenytoin [32]. The risk is lower for chronic users.

**Anabolic steroids and adrenal insufficiency**. According to Bleecker, Lee had been on and off anabolic steroids that he combined with diuretics to prevent steroid-induced fluid retention [4]. While off steroids, Bleecker suggested that he may have developed adrenal insufficiency. It is unclear what steroids he might have used and whether they may have suppressed adrenal function leading to adrenal insufficiency while off them, as performance-enhancing steroids usually have androgenic properties. However, chronic administration of opiates has the potential to cause secondary adrenal insufficiency [33] while phenytoin accelerates the metabolism of cortisol [34] and the combination of both may exacerbate any underlying predisposition to adrenal insufficiency. As indicated above, adrenal insufficiency is a further cause of hyponatraemia.

**Kidney dysfunction**. Lee appeared to have had an episode of AKI (i.e. an acute decrease in the kidney function of glomerular filtration) at the time of the first cerebral oedema episode that later recovered. This is unusual for an apparently healthy person, but diuretics may decrease kidney function through hypovolemia (i.e. loss of sodium in the urine). AKI may interfere with several kidney functions, including water excretion.

**Exercise**. Exercise-associated hyponatraemia is defined as hyponatraemia occurring during or up to 24 h after prolonged physical activity [35]. It has been mainly observed in endurance athletes in long events such as marathons. It is thought to depend on excess water intake when ADH is suboptimally suppressed because of intense exercise itself, nausea and/or vomiting, hypoglycaemia, pain, or release of muscle-derived interleukin-6 and may be exacerbated by NSAIDs [31]. Lee's exercise while actively acting out some scenes in his last day of life does not appear to qualify as a single trigger for exercise-associated hyponatraemia, it could have been a further contributing factor on top of those already discussed.

## Summary of the evidence

The answer to the question of whether Lee had factors predisposing to hyponatraemia is yes, not only one, but multiple factors that predispose to hyponatraemia:

- High chronic fluid intake.
- Factors that acutely increase thirst and water intake: marijuana, plus evidence that he was repeatedly drinking water on the day of his death.
- Factors that decrease the ability of the kidney to excrete a water load because they either increase ADH secretion or impair the collecting tubular response to ADH: prescription drugs (diuretics, NSAIDs, opioids, antiepileptic drugs), alcohol, chronic low solute intake, potentially an acute decrease in glomerular filtration rate such as that observed in May 1973, and exercise.

**FIGURE 2:**
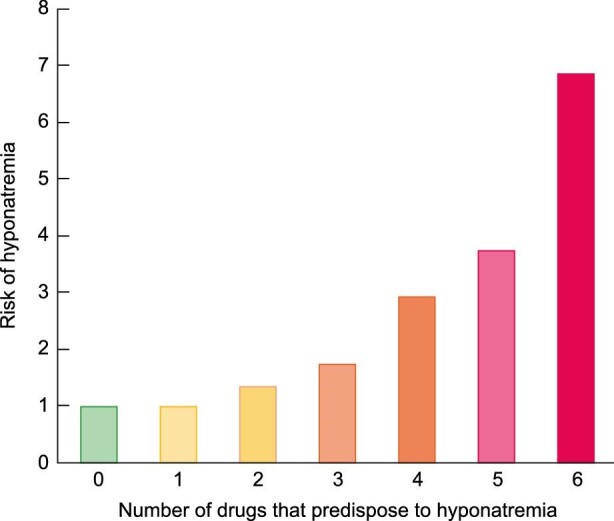
Prescription drugs and risk for hyponatraemia. A higher number of prescription drugs associated with hyponatraemia further increases the risk of hyponatraemia in patients with other predisposing factors. Bar graph built using data from reference [31].

In summary, Lee had multiple risk factors predisposing to hyponatraemia resulting from interference with water homeostasis mechanisms that regulate both water intake and water excretion (Table 3, Figure 1), a clinical presentation consistent with hyponatraemia and a necropsy finding of cerebral oedema which is the cause of death in severe hyponatraemia. This predisposition may have caused pre-existing asymptomatic hyponatraemia, which is associated with a high risk for the development of worsening hyponatraemia with altered mental status [36]. Pre-existing hyponatraemia was found in over 70% of all patients admitted with symptomatic hyponatraemia and represented the most common risk factor identified. Additionally, in patients with a baseline risk of hyponatraemia due to other factors, an increase in the number of prescription drugs that predispose to hyponatraemia synergistically increases the risk of hyponatraemia (Figure 2) [31]. Thus, even if some of the risk factors identified in the present report were not actually present or had a mild influence by themselves, the presence of multiple risk factors may explain the sequence of events that led to Lee’s demise.

## CONCLUSIONS

In conclusion, we hypothesize that Bruce Lee died from a specific form of kidney dysfunction: the inability to excrete enough water to maintain water homeostasis, which is mainly a tubular function. This may lead to hyponatraemia, cerebral oedema and death within hours if excess water intake is not matched by water excretion in urine, which is in line with the timeline of Lee’s demise. Given that hyponatraemia is frequent, as is found in up to 40% of hospitalized persons [37] and may cause death due to excessive water ingestion even in young healthy persons, there is a need for a wider dissemination of the concept that excessive water intake can kill. The fact that we are 60% [38] water does not protect us from the potentially lethal consequences of drinking water at a faster rate than our kidneys can excrete excess water. Ironically, Lee made famous the quote ‘Be water my friend’, but excess water appears to have ultimately killed him.

## Data Availability

This article is based on publicly available information that we could not verify.
